# Linoleic Acid—A Feasible Preventive Approach for Visceral Leishmaniasis

**DOI:** 10.3389/fnut.2021.649025

**Published:** 2021-02-26

**Authors:** Sheetal Saini, Ambak Kumar Rai

**Affiliations:** Department of Biotechnology, Motilal Nehru National Institute of Technology Allahabad, Prayagraj, India

**Keywords:** visceral leishmaniasis, malnutrition, ω-6 polyunsaturated fatty acid, linoleic acid, protective immunity

## Introduction

Kala-azar, also known as visceral leishmaniasis (VL), remains one of the top parasitic diseases and is fatal if left untreated in over 95% of cases. The condition is characterized by irregular fever bouts, weight loss, hepato-splenomegaly (enlargement of the spleen and liver) and anemia. Along with Sudan, Ethiopia, and Brazil, the Indian subcontinent contributes to ~90% of the global burden of visceral leishmaniasis (VL) (1). Post kala-azar dermal leishmaniasis (PKDL), a clinical complication of VL, where infection reoccurs in the dermal region after successful treatment, is also a challenge. The presence of the *Leishmania* parasite in the cutaneous region may serve the purpose of a reservoir for the consistent dissemination of disease. Therapeutic vaccines and newer potential drugs are under development/research, but unfortunately, we have nothing in hand. Thus, there is still a lot that needs to be done to achieve the absolute eradication of the disease. Prevalent malnutrition and resultant inadequate immune response are the practical limitations restricting the elimination of VL from endemic regions. Consequently, there is a need to look for newer methods to condition the immune system for a protective cellular response against *Leishmania* infection in endemic areas as a preventive measure.

Malnutrition is always considered as a possible risk factor for the advancement of VL (2, 3). It is associated with reduced immune response and increased visceralization of the parasite (4). Linoleic acid (LA) is an essential fatty acid (EFA) for humans due to the unavailability of fatty acid desaturase (FAD)-12. This enzyme is present in plants and thus, for humans, plant-derived diets are the primary source of LA. Arachidonic acid (AA, ω-6) is the derivative of LA and is known to set a background for instant innate immune response with its metabolites, i.e., eicosanoids. Humans can acquire arachidonic acid (AA, ω-6) either from animal based-diet or FAD-6 and FAD-5 synthesize AA from dietary LA (5). The low levels of LA are also reported in individuals suffering from malnutrition (6, 7). The inadequate dietary supply of LA is very much possible in endemic areas. We have also found low levels of LA in VL patients' serum compared to healthy individuals (8). Furthermore, it is established that the deficiency of EFA (i.e., LA) causes loss of water from the skin resulting in dark and patchy skin, which are the hallmark symptoms of VL disease (9, 10). This could be one of the plausible reasoning to explain skin darkening in VL patients. Malnutrition is reported to lead a relative increase in anti-inflammatory eicosanoids as compared to pro-inflammatory eicosanoids, which contributes to the compromised innate immune response against *Leishmania* infection (11).

## *Leishmania* Infection and ω-6 Polyunsaturated Fatty Acids (PUFA)

The pathogenesis of VL significantly depends upon macrophage (mϕ)—*Leishmania* interactions and further their encounter with T cells. As major components of the cellular membrane, PUFA plays a pivotal role in maintaining the membrane fluidity, which is essential for appropriate antigen presentation to T cells and modulate inflammatory and immune responses (12). Classically, human immune/inflammatory cells (mϕ, neutrophils, mast cells, etc.) are high in ω-6 PUFA compared to ω-3 (13). LA is converted to AA and stored in the plasma membrane of the cells, especially immune cells like tissue mϕ, dendritic cells, and neutrophils (14). Precisely, AA is positioned at the 2-acyl motif of membrane phospholipids (mainly phosphatidylcholine, phosphatidylethanolamine and phosphatidylinositol). Mϕ enriched with PUFAs, especially AA (ω-6) showed ~50% enhancement of phagocytic and adhesion capacity (15). LA also evokes superoxide release from neutrophils and mϕ (16), which is essential for eliminating the *Leishmania* parasite (17). LA is the precursor of ω-6 PUFAs in mammals and their deficiency at the cellular level impairs cell-to-cell interaction by modifying cell adhesion (18). This deficiency could cause improper immunological synapse formation, leading to compromised antigen presentation by antigen-presenting cells and inadequate lymphocyte activation (19), which may disseminate the disease.

AA viz. derivative of LA is converted to various metabolites belonging to either anti-inflammatory or pro-inflammatory classes, collectively called eicosanoids due to a 20-carbon (eicosa) precursor. These are the critical factors in inflammation and modulate the immunopathogenesis of various infections and auto-immune disorders (20). Among all eicosanoids, the effect of prostaglandin E2 (PGE2) and leukotriene B4 (LTB4) have been studied most widely. Immunologically, prostaglandins (PGs) are considered as anti-inflammatory and leukotrienes (LTs) as pro-inflammatory. PGE2 suppresses immune cells' proliferation, inhibits NK cell activity, upregulates IL-10 release by mϕ (21, 22) and reduces the production of Th1 cytokines (IFN-γ, IL-2, TNF-α, IL-1, and IL-6) by Th1 cells (23). On the other hand, LTB4 increases vascular permeability, enhances ROS generation and promotes NK cell activity (16). LTB4 enhances pro-inflammatory mediators (TNF, IL-1, IL-6, IL-2, IL-12, IFN-γ, and iNOS) and loss of 5-lipoxygenase (LTB4 final product) leads to decreased phagocytosis of by mϕ and neutrophils (24, 25).

**Figure 1:**
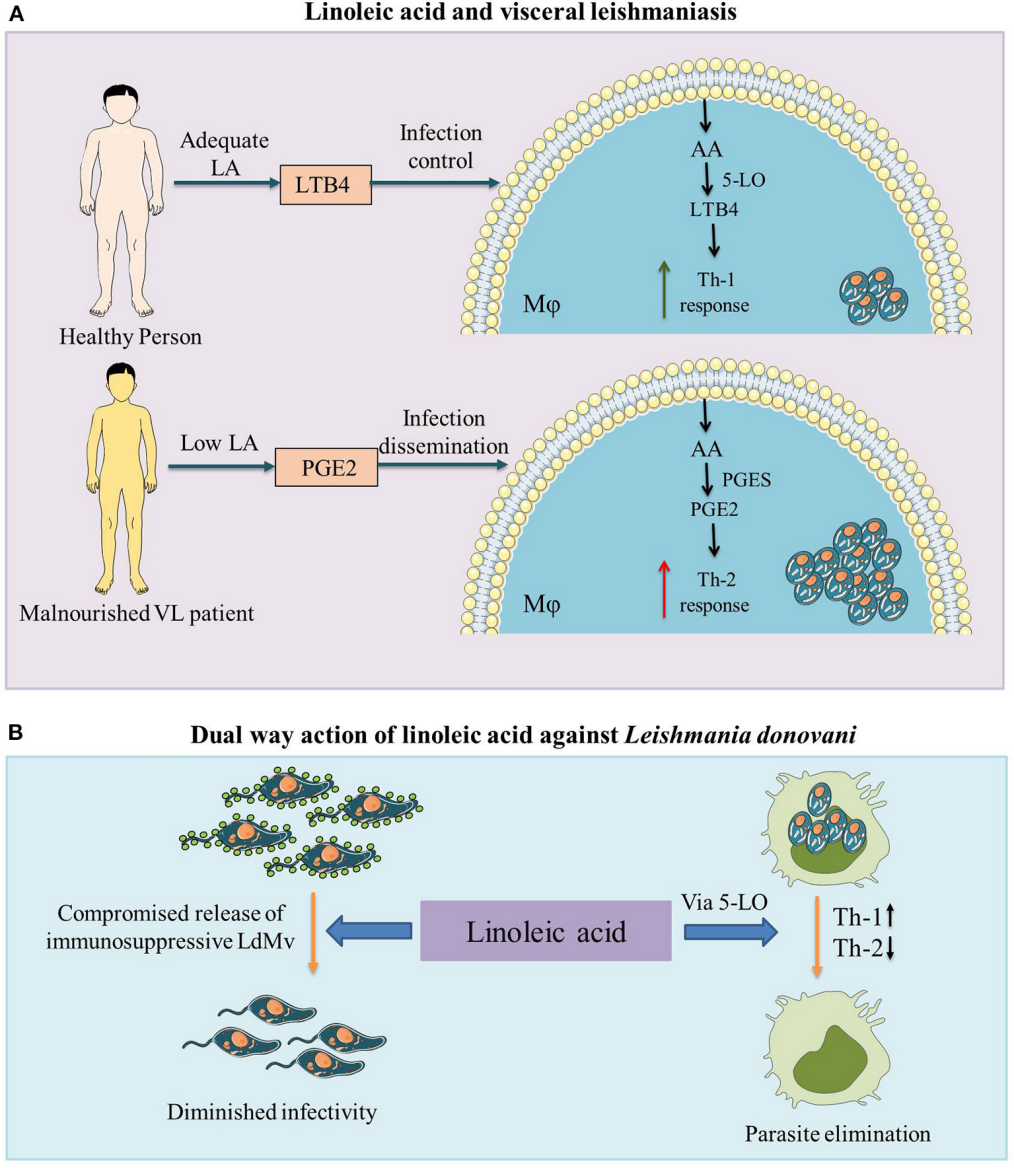
The role of linoleic acid against visceral leishmaniasis (VL) infection. **(A)** Malnutrition is associated with VL and in malnourished individuals as well as VL patients, low levels of linoleic acid (LA) are observed (7, 8). Malnutrition leads to a relative increase in anti-inflammatory prostaglandin E2 (PGE2) compared to pro-inflammatory leukotriene B4 (LTB4) (11). Higher levels of PGE2 with lower levels of LTB4 create an immunosuppressive environment (upregulated Th2 response) in the host, which leads to the advancement of VL infection. **(B)** Linoleic acid plays a dual way protective role against *Leishmania donovani* infection (i) by inhibiting the release of *Leishmania donovani* derived microvesicles (LdMv) from promastigote form of the parasite (36) and (ii) by promoting the protective Th-1 type pro-inflammatory (via the 5-lipoxygenase (5-LO) pathway and suppressing the Th-2 type anti-inflammatory immune response (8). LA, linoleic acid; LTB4, leukotriene B4; AA, arachidonic acid; 5-LO, 5-lipoxygenase; Mϕ, macrophage; VL, visceral leishmaniasis; PGE2, prostaglandin E2; PGES, prostaglandin E2 synthases; LdMv, *Leishmania donovani* derived microvesicles.

During leishmaniasis, the *Leishmania* parasite induces PGE2 generation in host mϕ and aid parasite survival (26, 27). *L. donovani* exploits the PGE2/EP2 pathway to reduce protective cytokines (TNF-α and IL-17) (28) and, along with arginase-I and TGF-β, is known to create a favorable immunosuppressive microenvironment for *L. amazonensis* dissemination (29). Sandfly (*L. longipalpis*) saliva is known to create a suppressive atmosphere in mϕ by inducing PGE2 production and favors *L. infantum* infection (30, 31). On the other hand, LTB4 formation is required for *L. amazonensis* elimination from mϕ (32). LTB4 via BLT1 receptor increases ROS generation and potentiates mϕ leishmanicidal activity. The decreased expression of the BLT1 receptor after *Leishmania* infection is indicative of a parasite escape mechanism for the chronic and sustained disease (33). Among other experimental models, the Syrian golden hamster (*Mesocricetus auratus*) is regarded as the best to investigate the immunopathogenesis in VL (34, 35). We have demonstrated the organ-specific role of LTB4 and PGE2 in experimental VL (*L. donovani* infected *M. auratus*). 5-lipoxygenase (final product LTB4) was prominent in the liver, which contained the parasitic load and the spleen showed upregulated expression of PGE2 synthases (final product PGE2) along with uncontrolled parasite burden (25). The dietary precursor of these eicosanoids, i.e., LA showed a protective response against VL infection in pre-clinical studies. LA inhibited the Th-2 response and promoted Th-1 response, resulting in significantly low *L. donovani* infection in mϕ (8). LA also reduced the release of immunosuppressive extracellular vesicles (microvesicles specifically) from *L. donovani* parasite (36). Taken together, LA plays a dual-way protective role in the immune response against *L. donovani* infection, firstly by inhibiting the release of *Ld*Mv and secondly promoting the Th-1 type immune response via the 5-LO pathway (Figure 1).

## Discussion

Despite the established protective role of LBT4 in VL, the possibilities of their therapeutic applications are limited due to their transient nature and cost issues. Thus, instead of using eicosanoids, LA, their dietary precursor, may have a beneficial role in disease containment. Higher levels of LTB4 but not PGE2 have been observed in the serum of LA supplemented healthy individuals (37). Varying concentrations of LA are already present in edible oils (Safflower > 75%; Sunflower > 60%; Soybean > 50%; Sesame > 40%, Rice bran > 30%; Groundnut > 25%; Peanut > 15%, Mustard > 10%, Olive ~10% and Coconut ~1%). There are no known side effects of LA supplementation in humans. On the contrary, LA intake is inversely associated with coronary heart disease risk. Summarily, a shift in dietary habits from LA-poor oils to LA-rich oils (safflower, sunflower, sesame, etc.) may have beneficial effects on disease containment in endemic areas.

## Author Contributions

SS drafted and wrote the manuscript. AR critically revised and edited the manuscript for important intellectual content. Both authors contributed to the article and approved the submitted version.

## Conflict of Interest

The authors declare that the research was conducted in the absence of any commercial or financial relationships that could be construed as a potential conflict of interest.

## Abbreviations

VL: Visceral Leishmaniasis
LA: Linoleic acid
*Ld*: *Leishmania donovani*
AA: Arachidonic acid
PUFA: Polyunsaturated fatty acids
EFA: Essential fatty acid
5-LO: 5-lipoxygenase
LTB4: Leukotriene B2
PGE2: Prostaglandin E2
Macrophages: Mϕ
*Ld*Mv: *Leishmania donovani*. derived microvesicles.
